# Opposite Responses of Interferon and Proinflammatory Cytokines Induced by Human Metapneumovirus and Respiratory Syncytial Virus in Macrophages

**DOI:** 10.3390/pathogens14070694

**Published:** 2025-07-14

**Authors:** Iván Martínez-Espinoza, Antonieta Guerrero-Plata

**Affiliations:** Department of Pathobiological Sciences, School of Veterinary Medicine, Louisiana State University, Baton Rouge, LA 70803, USA

**Keywords:** HMPV, RSV, macrophages, MDMs, proinflammatory, innate immunity, IFN, cytokines

## Abstract

Macrophages are a principal pulmonary source of type I and III interferons (IFNs), initiating and coordinating the early antiviral response to respiratory viral infections. Yet the contribution of macrophage-derived IFNs to host defense during human metapneumovirus (HMPV) infection remains poorly defined. Here, we use human primary monocyte-derived macrophages (MDMs) and THP-1-derived macrophages to analyze the IFN responses induced by HMPV compared to its closely related human pneumovirus, respiratory syncytial virus (RSV). We show that HMPV induced a robust response of type I and type III IFNs and ISGs, whereas RSV elicited only a modest, delayed IFN response despite strong IRF activation; instead, RSV preferentially activates NF-κB and exhibits a pronounced proinflammatory cytokine output. Our results highlight the role of macrophages as key modulators of the IFN and proinflammatory responses during HMPV and RSV infection.

## 1. Introduction

In the lungs, alveolar macrophages are essential producers of interferons (IFNs) during RNA viral infections [1]. These cells are strategically positioned in the respiratory tract as first responders to viral invasion, rapidly sensing viral pathogens and secreting IFNs and other cytokines to combat infection [1,2,3,4]. Commonly, they secrete type I IFNs, such as IFN-β, followed by multiple IFN-α subtypes [1,5], yet other less common isoforms of IFNs, such as IFN-ε and -ω, have not been described. Additionally, they induce type III IFNs, mainly IFN-λ1 and 2/3 [6,7,8,9]; together, IFNs induce an antiviral state in infected and neighboring cells by inducing the expression of IFN-stimulated genes (ISGs), limiting viral replication and spread [2,10,11]. Despite the recognized effect of the IFN responses in human pneumoviruses [12,13,14,15,16,17,18,19], the contribution of macrophages to the IFN responses induced by human metapneumovirus (HMPV) has not been comprehensively explored.

HMPV is a major cause of lower respiratory tract infections, particularly in young children, the elderly, and immunocompromised individuals, and is similar to respiratory syncytial virus (RSV), the closest related member of the *Pneumoviridae* family [20,21,22,23,24,25,26,27,28,29,30,31]. Both viruses are enveloped, negative-sense, single-stranded RNA viruses that primarily infect airway epithelial cells [32,33]. However, despite their structural and clinical similarities, these viruses elicit distinct immune responses in the infected host [34,35]. Moreover, RSV has been more extensively studied, while the immune response to HMPV remains less characterized [14,36,37].

This study used human monocyte-derived macrophages (MDMs) as an in vitro model to determine the IFN response induced by HMPV in a comparative analysis with RSV. We tracked the timing and strength of HMPV-triggered type I and III IFN responses, including ISG expression and IFN-related proinflammatory cytokines. Also, an engineered model of THP-1-derived macrophages was used to monitor the NF-κB and IRF activation. Overall, these findings defined that HMPV shapes the IFN response in macrophages, while RSV elicited mostly a proinflammatory response in these cells.

## 2. Materials and Methods

### 2.1. Cell Culture

Primary monocyte-derived macrophages (MDMs) were differentiated from monocytes isolated from peripheral blood mononuclear cells (PBMCs) through Ficoll-Paque (Cytiva, Marlborough, MA, USA) density gradient separation and subsequent adherence. PBMCs were obtained from buffy coats provided by the Our Lady of the Lake Blood Donor Center, with no access to donors’ identifiers. Their use does not constitute human subject research under the U.S. Department of Health and Human Services (HHS) human subject regulations (45 CFR Part 46) [38] and is therefore exempt from Institutional Review Board oversight and informed consent requirements. Monocytes were cultured in RPMI 1640 (HyClone, Logan, UT, USA) supplemented with 10% fetal bovine serum (FBS) (HyClone, Logan, UT, USA) and 100 ng/mL of granulocyte-macrophage colony-stimulating factor (GM-CSF) (Peprotech, Cranbury, NJ, USA) for 7 days with fresh supplemented media changes every two days.

The THP-1 Dual ™ cell line (Invitrogen, San Diego, CA, USA) was cultured in RPMI 1640 (HyClone, Logan, UT, USA) supplemented with 10% FBS (Biowest, Riverside, MO, USA) and 1% penicillin-streptomycin (HyClone, Logan, UT, USA). Cells were maintained in incubation with 5% CO_2_ at 37 °C and were used for experiments between passages 5 and 12. THP-1 Dual™ cells express reporter genes for NF-κB and IRFs. THP-1 cells were differentiated into macrophages by the addition of 100 ng/mL of phorbol 12-myristate 13-acetate (PMA) (Millipore Sigma, Burlington, MA, USA), as previously reported [39].

LLC-MK2 cells (ATCC, CCL-7, Manassas, VA, USA) and Hep2 cells (ATCC CCL 23, Manassas, VA, USA) were propagated in MEM/EBSS medium (HyClone, Logan, UT, USA) enriched with 10% FBS (Biowest, Riverside, MO, USA) and 1% penicillin-streptomycin (HyClone, Logan, UT, USA).

### 2.2. Virus Stocks

The HMPV strain CAN97-83 was propagated in LLC-MK2 cells in MEM containing 1 μg of trypsin/mL (Worthington Biochemicals, Lakewood, NJ, USA) and purified sucrose, as previously reported [12,40]. HMPV pools were titrated (PFU/mL) by a combined method of methylcellulose plaque assay and cell-based immunoassay in LLC-MK2 cells, as previously described [34]. RSV strain A2 was purchased (ATCC, Manassas, VA, USA) and purified by a sucrose gradient, as previously reported [34,41]. The RSV viral titer (PFU/mL) was determined by a methylcellulose plaque assay on HEp-2 cells, as described elsewhere [42].

### 2.3. Viral Infection

Macrophages were plated at 1.5 × 10^5^ cells per well in a 24-well plate and infected with HMPV or RSV. Based on previous studies characterizing the infection in macrophages [39], the cells were infected at a multiplicity of infection (MOI) of 1.0. For HMPV, the cells were infected in the presence of 1 μg/mL of trypsin (Worthington Biochemical, Lakewood, NJ, USA) in plain RPMI 1640 medium. After 2 h of RSV or HMPV adsorption, the inoculum was removed and complemented with RPMI medium [RPMI/10% FBS (Biowest, Riverside, MO, USA) and 1% penicillin-streptomycin (HyClone, Logan, UT, USA)].

### 2.4. RNA Extraction and Quantitative Real-Time Reverse Transcription PCR (qRT-PCR)

Cell samples were lysed using the lysis buffer supplied with the RNeasy Plus kit (Qiagen, Germantown, MD, USA), and the total RNA was purified according to the kit protocol. RNA concentrations were quantified, adjusted to equal amounts, and then used for first-strand cDNA synthesis with qScript cDNA SuperMix (5×) (Quanta Bio, Beverly, MA, USA), according to the manufacturer’s instructions. The resulting cDNA was amplified with PowerTrack SYBR Green Master Mix (Thermo Fisher Scientific, Waltham, MA, USA) on the QuantStudio™ 12k PCR system (Applied Biosystems, Foster City, CA, USA). Primers for IFNA2, IFNB, IFNE, IFNW, IFNL1, IFNL2/3, ISG15, OAS1, MX1, IRF7, and GAPDH were obtained from Integrated DNA Technologies (IDT, Coralville, IA, USA). Relative gene expression was determined by the ΔΔCT method and normalized to the GAPDH gene as the endogenous control. All data were analyzed with QuantStudio™ 12k Flex Software Version 1.3.

### 2.5. Human Interferon and Related Inflammatory Cytokine Release Assay

Culture supernatants collected from infected MDMs were analyzed for type I (IFN-α, IFN-β, and IFN-ω), type II (IFN-γ), and type III IFNs (IFN-λ1/2/3), along with related proinflammatory cytokines (IL-1α, TNF-α, IL-6, and IP-10) utilizing the VeriPlex Human Interferon 9-Plex ELISA kit (PBL Assay Science, Piscataway, NJ, USA). This ELISA kit employs a sandwich immunoassay format with nine discrete capture-antibody spots on each well. Following sample incubation, biotinylated detection antibodies and an HRP-streptavidin conjugate generated a chemiluminescent signal proportional to each IFN abundance. Signal intensities for the nine cytokine-specific spots, plus built-in internal controls, were then imaged and quantified on the Azure 600 (Azure Biosystems, Dublin, CA, USA) imaging system using the binning 1 × 1, with an exposure time of 30 s. After exposure, the image was converted into a negative. Spot intensities were analyzed using Q-View™ Software version 3.131 according to the manufacturer’s recommendations.

### 2.6. NF-κB and IRF Activity

For the quantification of the Nuclear Factor kappa-light-chain-enhancer of activated B cells (NF-κB) and Interferon Regulatory Factor (IRF) activation, we used human monocytic THP1-Dual™ cells. These cells are engineered to express two distinct reporter proteins, secreted embryonic alkaline phosphatase (SEAP) under the control of an NF-κB responsive promoter and Lucia luciferase under the control of an interferon regulatory factor (IRF) responsive promoter to monitor IRF activity, which includes IRF 1/3/5/7. This allows for the simultaneous, quantitative monitoring of NF-κB and IRF pathway activation in response to the viral infection. Cells were differentiated into macrophages and then infected with either HMPV or RSV at an MOI of 1.0. Uninfected cells served as control. Supernatants were collected at 24 and 48 h after infection to assess NF-κB activation using the Quanti-Blue™ assay (Invitrogen, San Diego, CA, USA) and IRF activation using the QUANTI-Luc™ 4 luciferase assay (Invitrogen, San Diego, CA, USA). Reporter signals were measured using a plate reader, following the manufacturer’s guidelines. SEAP activity was quantified by measuring optical density (OD) at 650 nm, while Lucia luciferase signals were detected using end-point luminescence readings with a 0.1 s integration time.

### 2.7. Statistical Analysis

Two-way ANOVA (Analysis of Variance) at a 0.05 significance level was used to compare differences among experimental groups, followed by an appropriate post hoc test to correct for multiple comparisons. All analyses were conducted with GraphPad Prism 10.4.2 (GraphPad Software, Boston, MA, USA).

## 3. Results

### 3.1. Expression of Type I and Type III IFNs in MDMs Infected with HMPV and RSV

**Figure 1:**
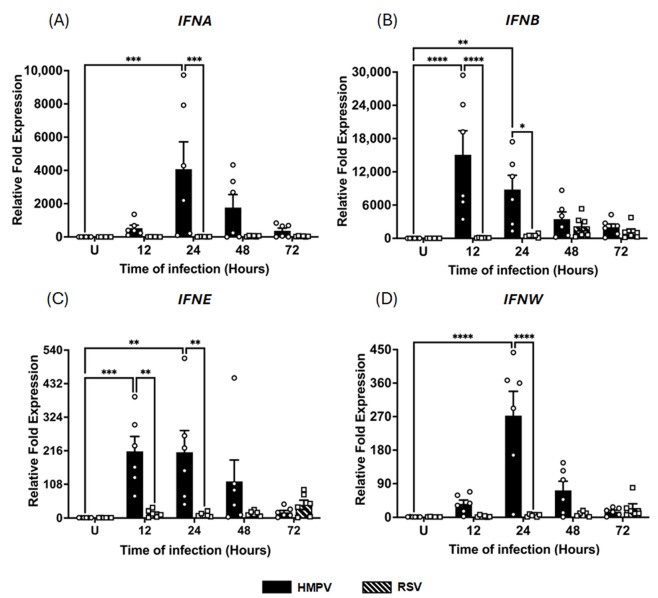
IFN-I response induced in macrophages by HMPV and RSV infection. MDMs were infected with HMPV or RSV at an MOI of 1.0. Cell lysates were collected at 12, 24, 48, and 72 h after infection and analyzed by RT-qPCR to determine expressions of (**A**) IFN-α (IFNA), (**B**) IFN-β (IFNB), (**C**) IFN-ε (IFNE), and (**D**) IFN-ω (IFNW). The bar graphs with scatter plots represent the mean ± SEM (*n* = 6). U = uninfected control cells. Statistical differences were calculated using ordinary two-way ANOVA followed by a Šidák multiple comparison test. * *p* < 0.05, ** *p* < 0.01, *** *p* < 0.001, **** *p* < 0.0001.

Given the established role of macrophages as a key source of IFNs during viral infections in the lungs with RNA viruses [1], we sought to investigate the type I and III IFN responses triggered by HMPV infection and compare them to those induced by RSV. MDMs were infected with either HMPV or RSV at an MOI of 1.0. Cell lysates and supernatants were collected at 12, 24, 48, and 72 h after infection to determine virus-specific patterns and kinetics of IFN induction in macrophages. Total RNA was extracted, and gene expression was analyzed by RT-qPCR. We assessed the expression of type I IFNs, including IFN-α, IFN-β, IFN-ε, and IFN-ω, and type III IFNs (IFN-λ1, IFN-λ2/3, and IFN-λ4). As shown in Figure 1A, our results indicate that HMPV infection led to a robust and early upregulation of IFN-α gene expression at 12 h (525 ± 183.8-fold increase, relative to uninfected (U) control cells) with a peak of a 4074 ± 1643.7-fold increase at 24 h, followed by a gradual decline to 1768 ± 782.1-fold at 48 h and a sustained expression of 371 ± 158.6-fold at 72 h. In contrast, RSV induced less IFN-α expression in a delayed manner, reaching its peak at 48 h with a magnitude of a 43 ± 14.9-fold increase and a sustained expression through 72 h (34 ± 10.1-fold). A similar trend was observed for IFN-β, with HMPV triggering an increased expression (17,000 ± 4348.2-fold) at 12 h. In contrast, RSV induced a much lower and delayed response of about 2000 ± 816.6-fold at 48 h (Figure 1B). Regarding the expression of IFN-ε and IFN-ω, HMPV also drove significantly stronger responses than RSV. As shown in Figure 1C, HMPV induced a 214 ± 47.9-fold increase in IFN-ε, peaking as early as 12 h, whereas RSV induced a weaker and delayed response of a 40 ± 14.7-fold increase with the maximum expression at 72 h. For IFN-ω, HMPV induced a peak at 24 h of 272 ± 65.1-fold; its expression declined rapidly to 72 ± 24.6-fold at 48 h and 15 ± 4.0-fold at 72 h. On the other hand, RSV-infected cells expressed an IFN-ω pattern similar to that of IFN-ε, showing a marginal expression of 2.5 ± 1.0-fold at 12 h, increasing to a maximum of 25 ± 11.16-fold at 72 h (Figure 1D). Overall, these data indicate that HMPV elicits a more robust IFN response than RSV in macrophages.

**Figure 2:**
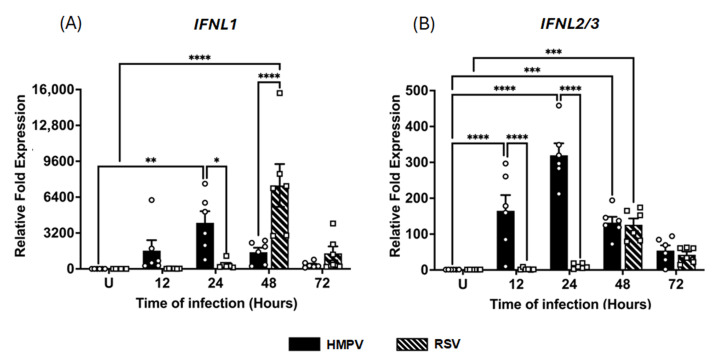
IFN-III response induced in macrophages by RSV and HMPV infection. MDMs were infected with HMPV or RSV at an MOI of 1.0. Cell lysates were collected at 12, 24, 48, and 72 h after infection and analyzed by RT-qPCR to determine expression amounts of (**A**) IFN-λ1 (IFNL1) and (**B**) IFN-λ2/3 (IFNL2/3). The bar graphs with scatter plots represent the mean ± SEM (*n* = 6). U = uninfected control cells. Statistical differences were calculated using ordinary two-way ANOVA followed by a Šidák multiple comparison test. * *p* < 0.05, ** *p* < 0.01, *** *p* <0.001, **** *p* < 0.0001.

The assessment of type III IFN expression indicated that the induction of IFN-λ isoforms was also markedly different between the two viral infections. As shown in Figure 2A, IFN-λ1 expression peaked at 24 h in HMPV-infected macrophages with a 4112 ± 1035.4-fold increase, decreasing to 364 ± 108.7-fold at 72 h. In RSV-infected cells, the IFN-λ1 expression was relatively low at 12 h (19 ± 4.9-fold) but increased sharply to a maximum expression at 48 h, reaching levels of 7444 ± 1907.4-fold, even higher than those induced by HMPV. In contrast, a distinct expression pattern was shown for IFN-λ2/3 (Figure 2B). HMPV triggered a rapid induction at 12 h (165 ± 43.6-fold) with a peak of 319 ± 33.3-fold at 24 h, gradually declining to 54 ± 14.3-fold at 72 h. On the other hand, compared to HMPV, RSV induced a reduced expression of IFN-λ2/3, showing a 2.5 ± 1.2-fold at 12 h and a peak of 126 ± 17.4-fold at 24 h, but its expression remained above the baseline levels (42 ± 8.8-fold) at 72 h. No expression of IFN-λ4 was induced by either RSV or HMPV in MDMs. Taken together, these data suggest that both RSV and HMPV can induce a strong response of IFN-III, although RSV induced a delayed kinetic profile compared to HMPV.

### 3.2. Induction of Interferon-Stimulated Gene (ISG) Response by HMPV and RSV in MDMs

**Figure 3:**
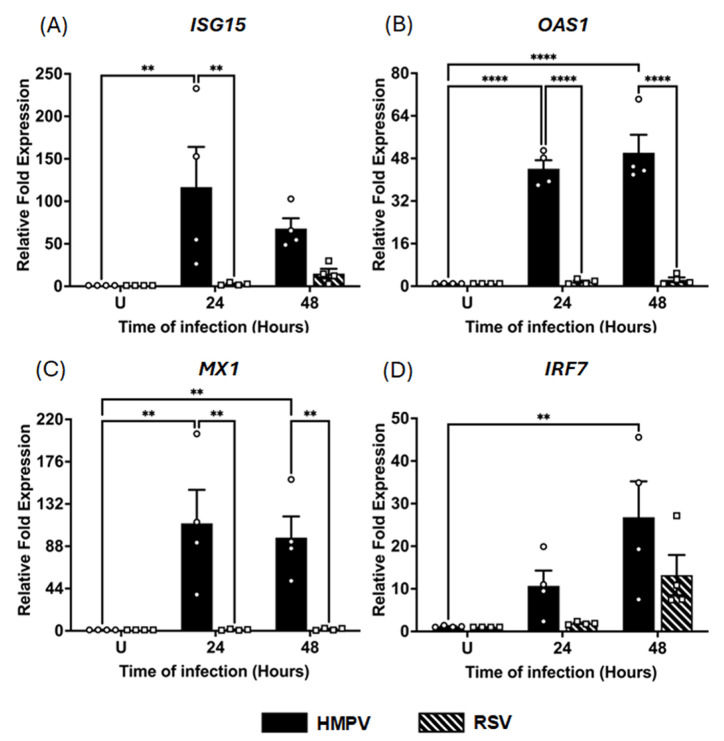
ISG induction in HMPV- and RSV-infected macrophages. MDMs were infected with HMPV or RSV at an MOI of 1.0 Cell lysates were collected at 24 and 48 h for analysis by RT-qPCR to determine gene expressions of (**A**) ISG15, (**B**) OAS1, (**C**) MX1, and (**D**) IRF7. The bar graphs with scatter plots represent the mean ± SEM (*n* = 4). U = uninfected control cells. Statistical differences were calculated using ordinary two-way ANOVA followed by a Šidák multiple comparison test. ** *p* < 0.01, **** *p* < 0.0001.

IFNs are known to induce a broad transcriptional program of ISGs [43,44,45], which can restrict viral replication and modulate the host immune response [45,46]. Therefore, based on the capacity of macrophages to express IFNs in RSV and HMPV infections, we next determined the effect of these pneumoviruses on the induction of ISGs in MDMs. The expression of ISG15, OAS1, MX1, and IRF7, implicated in the antiviral response against pneumoviruses [47,48,49,50], was assessed under the same experimental conditions as those above. As shown in Figure 3, HMPV infection induced a markedly stronger ISG response than RSV. Specifically, ISG15 expression (Figure 3A) was strongly increased by HMPV infection by 116 ± 47.2-fold at 24 h and decreased to 68 ± 12.1-fold at 48 h. In contrast, RSV induced a marginal 2.6 ± 0.7-fold increase at 24 h and a 15 ± 5.6-fold increase at 48 h. Similarly, OAS1, a key antiviral enzyme, was induced by HMPV to 44 ± 3.2-fold levels at 24 h and 50 ± 6.7-fold levels at 48 h. Regarding RSV, it induced a low expression of OAS1 of 1.6 ± 0.4-fold at 24 h, which increased to 2.4 ± 0.8-fold at 48 h (Figure 3B). For the induction of MX1, a GTPase with direct antiviral activity, HMPV triggered a robust increase of 112 ± 69.9-fold at 24 h, maintaining similar levels (97 ± 44-fold) at 48 h. In contrast, RSV failed to induce MX1 expression at either time point, remaining close to baseline levels (Figure 3C). Finally, as shown in Figure 3D, IRF7, a transcription factor that is essential for the amplification of the IFN response, was upregulated by HMPV with a 21 ± 8.3-fold increase at 48 h. In comparison, RSV induced a weaker response of 13 ± 4.7-fold at 48 h, with no significant induction at 24 h. These results indicate that unlike RSV, HMPV elicits a potent and sustained ISG response in MDMs, with a strong induction of antiviral effectors.

### 3.3. HMPV and RSV Differentially Induce IFN and Inflammatory Cytokine Release in MDMs

**Figure 4:**
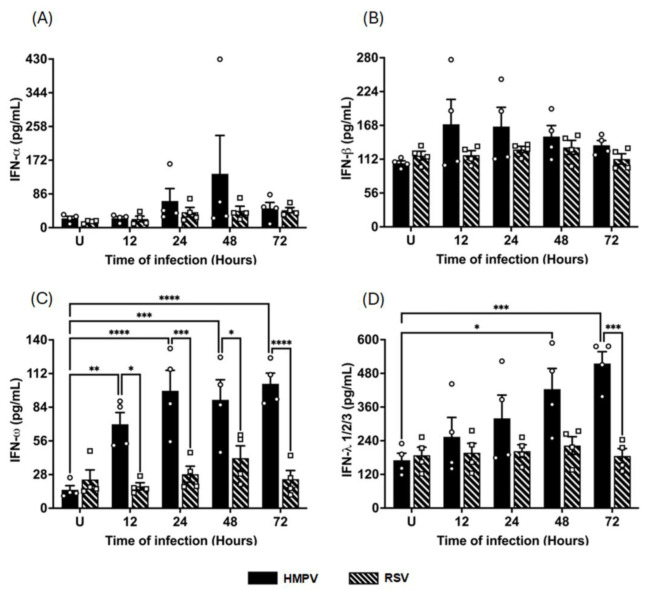
IFN release from HMPV- or RSV-infected macrophages. MDMs were infected with HMPV or RSV at an MOI of 1.0. Cell-free supernatants were collected at 12, 24, 48, and 72 h, and concentrations of (**A**) IFN-*α*, (**B**) IFN-*β*, (**C**) IFN-*ω*, and (**D**) IFN-*λ*1/2/3 were determined using VeriPlex Human Interferon 9-Plex. The bar graphs with scatter plots represent the mean ± SEM (*n* = 4). U = uninfected control cells. Statistical differences were calculated using ordinary two-way ANOVA followed by a Šidák multiple comparison test. * *p* < 0.05, ** *p* < 0.01, *** *p* < 0.001, **** *p* < 0.0001.

To determine whether the pattern of IFN secretion aligned with the observed transcriptional responses, we next quantified the levels of released type I, II, and III IFNs, as well as IFN-related proinflammatory cytokines (IL-1α, IL6, TNF- α, and IP-10) in the supernatants of infected macrophages. MDMs were infected with either HMPV or RSV at an MOI of 1.0, and supernatants were collected at 12, 24, 48, and 72 h after infection. Protein levels were quantified using the VeriPlex Human Interferon 9-Plex ELISA. As shown in Figure 4A, IFN-α levels induced by HMPV infection peaked at 137 ± 97.8 pg/mL by 48 h, remaining at 49 ± 15.2 pg/mL by 72 h, while RSV induced modest levels of IFN-α secretion, reaching a plateau of 40 ± 12.1 pg/mL at 24 h. Regarding the secretion of IFN-β, HMPV induced a small concentration that peaked at 12 h with an expression of 170 ± 41.1 pg/mL, representing an increase of 60% over the uninfected cells. RSV induced a negligible expression of IFN-β (Figure 4B). Data shown in Figure 4C indicates that IFN-ω was highly induced by HMPV in MDMs with an early expression at 12 h with 70 ± 9.7 pg/mL, reaching a peak between 24 and 72 h with concentrations around 100 pg/mL. With a similar pattern observed in the induction of IFN-α, RSV induced only a marginal amount of IFN-ω. As observed in Figure 4D, HMPV induced increasing concentrations of IFN-λ starting as early as 12 h with a concentration of 254 ± 68.9 pg/mL, reaching the maximum level of 515 ± 42.1 pg/mL at 72 h. IFN-γ was not detected in any of the tested samples.

**Figure 5:**
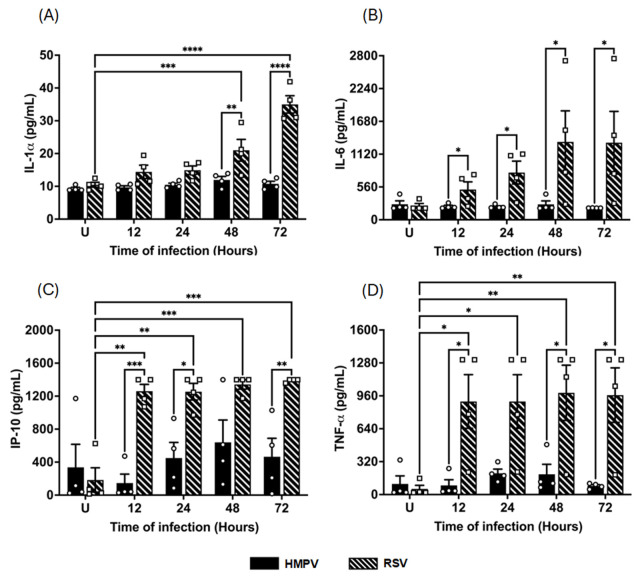
Cytokine release from HMPV- or RSV-infected macrophages. MDMs were infected with HMPV or RSV at an MOI of 1.0. Cell-free supernatants were collected at 12, 24, 48, and 72 h, and concentrations of (**A**) IL-1*α*, (**B**) IL-6, (**C**) IP-10, and (**D**) TNF-*α* were determined for analysis using VeriPlex Human Interferon 9-Plex. The bar graphs with scatter plots represent the mean ± SEM (*n* = 4). U = uninfected control cells. Statistical differences were calculated using ordinary two-way ANOVA followed by a Šidák multiple comparison test. * *p* < 0.05, ** *p* < 0.01, *** *p* < 0.001, **** *p* < 0.0001.

Nevertheless, RSV induced a more pronounced proinflammatory cytokine response than HMPV. We discovered this effect when measuring proinflammatory cytokines commonly associated with IFN responses, such as IL-1*α*, IL-6, IP-10 (CXCL10), and TNF-*α*. As shown in Figure 5A, IL-1*α* levels steadily increased during RSV infection compared to uninfected cells (10 *±* 0.7 pg/mL), reaching a peak of 35 *±* 2.6 pg/mL at 72 h. In contrast, HMPV did not induce IL-1*α*. A similar effect was observed in the induction of IL-6 (Figure 5B), where uninfected cells displayed a baseline concentration of 237 *±* 39.8 pg/mL, which was increased after RSV infection to concentrations exceeding 1330 *±* 529 pg/mL by 48–72 h. However, IL-6 was not induced by HMPV. Likewise, IP-10 and TNF-*α*, both known to be induced downstream from IFN signaling, were secreted at significantly higher levels during RSV infection. Data shown in Figure 5C indicate that IP-10 was highly induced by RSV as early as 12 h (1260 *±* 82.7 pg/mL), and those levels were sustained up to 72 h, as the last time point analyzed. HMPV did not induce significant levels of IP-10. Finally, the production of TNF-*α* in RSV-infected macrophages showed a similar induction pattern to that of IP-10, where TNF-*α* rapidly reached a plateau by 12 h with concentrations around 900 *±* 260.8 pg/mL. In contrast, HMPV induced lower concentrations (200 *±* 40.7 pg/mL) that peak at 24 h. Taken together, these data demonstrate that while HMPV infection triggers higher secretion of antiviral IFNs, RSV elicits stronger proinflammatory cytokine production in macrophages. This differential cytokine profile supports the idea that these two viruses activate distinct innate immune programs in macrophages, with HMPV favoring IFN-mediated antiviral defense and RSV driving a more inflammatory signature.

### 3.4. NF-κB and IRF Activation by RSV and HMPV in Macrophages

**Figure 6:**
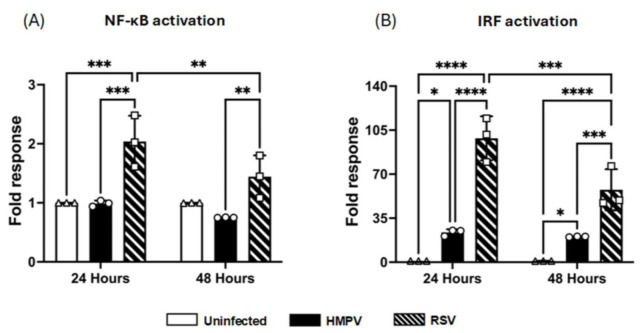
NF-κB and IRF activation in macrophages by RSV and HMPV. THP-1-derived-macrophages were infected with HMPV or RSV at an MOI of 1.0, and supernatants were collected at 24 and 48 h to assess (**A**) NF-κB using a colorimetric assay and (**B**) IRF activation by luminescence quantification. The bar graphs with scatter plots represent the mean ± SEM (*n* = 3). Statistical differences were calculated using two-way ANOVA followed by Tukey’s multiple comparison test. * *p* < 0.05, ** *p* < 0.01, *** *p* < 0.001, **** *p* < 0.0001.

Based on the above observations in which RSV-infected MDMs secreted higher levels of inflammatory cytokines, but HMPV infection was associated with stronger IFN production, we then analyzed the activation of key transcription factors such as NF-κB and IRFs that control the expression of cytokines. Specifically, NF-κB plays a central role in the induction of proinflammatory cytokines such as IL-1*α*, IL-6, IP-10, and TNF-*α*, while IRFs are critical for the transcription of IFNs and ISGs. We used macrophages derived from the THP1-Dual cell line, which express two reporter systems to identify the activation of both transcription factor families, including the secreted alkaline phosphatase (SEAP) as a readout for NF-κB activation and Lucia luciferase, which is expressed under an IRF-inducible promoter to monitor IRF activity, including IRF1, IRF3, IRF5, and IRF7. THP-1-derived macrophages were infected with RSV or HMPV at an MOI of 1.0. After infection, cell-free supernatants were collected at 24 and 48 h for their analysis. Our results demonstrated that RSV infection led to a significantly higher SEAP response at 24 h after infection than uninfected control cells, indicating significant NF-kB activation. This activation pattern persisted at 48 h, supporting the finding that RSV strongly activates proinflammatory signaling pathways. On the other hand, HMPV did not induce an activation of NF-κB in the infected cells (Figure 6A). Data analysis of the luciferase response indicates that RSV induced strong IRF activation (98 *±* 6.4-fold over the uninfected control) at 24 h after infection and remained high (57 *±* 6.0-fold) at the 48 h time point. Regarding HMPV, it also induced IRF activation. However, the response was weaker than that induced by RSV, observing an approximate 23 *±* 0.9-fold response at 24 h, which was sustained at 48 h (Figure 6B). Overall, these data suggest that RSV activates both NF-κB and IRF transcription factors, while HMPV activates mostly the IRF signaling in the infected macrophages.

**Figure 7:**
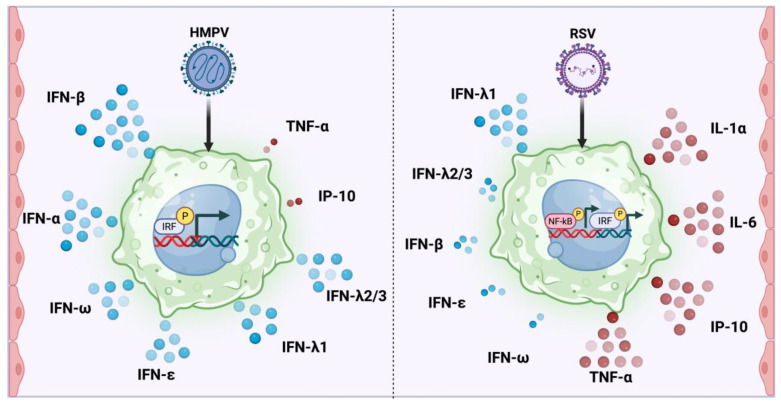
Differential induction of type I and III IFNs and inflammatory cytokines by HMPV and RSV in macrophages. HMPV infection in macrophages predominantly activates the IRF pathways and induces a robust expression of type I (IFN-α, IFN-β, IFN-ε, IFN-ω) and type III (IFN-λ1, IFN-λ2/3) IFNs, but generates a marginal production of TNF-α and IP-10. In contrast, RSV infection induces the activation of NF-κB and IRF and a strong proinflammatory response, characterized by high levels of IL-1α, IL-6, IP-10, and TNF-α, and a mild IFN response that includes the expression of IFN-λ1 and a low induction of IFN-β, IFN-ε, and IFN-ω. The expressions of IFN-γ and IFN-λ4 were undetected.

Based on the overall results, the differential activations of the antiviral and proinflammatory responses of macrophages to RSV and HMPV infection are summarized in Figure 7.

## 4. Discussion

The lung is constantly exposed to airborne pathogens, requiring rapid and localized immune responses to prevent infections. Alveolar macrophages are among the first immune cells to defend against viruses and are key producers of IFNs in the lung during RNA viral infections [1]. These IFNs initiate a broad antiviral program, restricting viral replication and coordinating the recruitment of additional immune effectors [43,44,45]. Although HMPV and RSV belong to the same family and share broad clinical and structural similarities, they activate different immune responses [34,51,52,53,54]. Previous studies in vivo have highlighted the dual role of alveolar macrophages in these infections: their depletion during HMPV infection has been shown to reduce disease severity, whereas macrophage removal in RSV infection leads to a worsened pathology and increased inflammation, indicating virus-specific roles in disease progression [55]. These contrasting outcomes underscore the need to better understand how macrophages contribute to antiviral signaling, particularly through type I and III IFN responses, which are key to controlling infection and modulating inflammation [56,57,58,59]. Given the importance of macrophages in the IFN production against RNA viruses [1] and the different immune responses between HMPV and RSV, we aimed to characterize the IFN response of human macrophages in HMPV infection and compare it to that of RSV as the precise role of macrophages in antiviral immunity against HMPV remains insufficiently characterized. We focused on differences in the kinetics, magnitude, and pattern of IFN induction, the antiviral response in these cells, and the activation of key transcription factors between these two closely related pneumoviruses.

The IFN response was differentially induced by RSV and HMPV in MDMs. We observed that HMPV provokes an early, high response of type I IFNs, with peak transcript levels appearing between 12 h and 24 h after infection and remaining through 48 h. This pattern is consistent with published work demonstrating that HMPV robustly stimulates IFN-β and IFN-λ production in macrophages and epithelial cells [14,60]. RSV, in contrast, induced only modest increases in the same type I IFN genes with a delayed response. This muted signature in RSV-infected cells might be related to the RSV non-structural proteins NS1 and NS2, which are known to antagonize RIG-I/MAVS signaling and block IRF3/7 activation [61,62,63,64,65], thereby suppressing the transcription of IFN-α and IFN-β in A549 cells and MDMs [9,66]. Our findings on the type III IFN response show that HMPV induced an early wave of IFN-λ1 and IFN-λ2/3, plateauing by 24 h and declining thereafter, mirroring the early type I IFN surge. RSV, however, displayed a delayed expression in which IFN-λ1 and IFN-λ2/3 induction remained weak at early time points but rose sharply at 48 h, with RSV surpassing the IFN-λ1 levels induced by HMPV. Functionally, the predominance of IFN-λ1 over IFN-λ2/3 late in RSV infection could influence epithelial barrier protection, as IFN-λ1 has been reported to act more potently on the airway epithelium [67]. Together, these results underscore that closely related viruses exploit distinct temporal strategies to modulate macrophage IFN outputs, with a rapid and potent induction by HMPV compared to RSV.

ISGs are the effector arm of the IFN system: once induced, they encode enzymes and restriction factors that block every step of the viral life cycle and amplify innate signaling [44,45]. Even though macrophages are not the primary site of pneumovirus replication like epithelial cells are [68,69,70,71], measuring ISG induction in these sentinel cells is critical because (i) macrophage-derived ISGs create an antiviral “paracrine shield” for neighboring epithelial cells and (ii) several ISGs, such as ISG15 and IRF7, feedback to fine-tune cytokine production in the lung [43,44,45,47,72]. Among the ISGs quantified here, ISG15, MX1, and OAS1 represent three mechanistically distinct antiviral effectors. ISG15 is a ubiquitin-like protein that can be conjugated (ISGylated) to viral or host proteins, destabilizing viral components, enhancing IFN signaling, and regulating cytokine secretion [72]. MX1 is a dynamin-like GTPase that assembles into oligomeric rings around incoming ribonucleoprotein complexes and blocks the viral transcription of numerous negative-strand RNA viruses [73]. OAS1 synthesizes 2ʹ-5ʹ-linked oligoadenylates that activate RNase L, leading to viral and cellular RNA degradation and halting replication [74]. Here, we show that HMPV drives a markedly stronger ISG induction than RSV. At 24 h post-infection, HMPV boosted ISG15, OAS1, MX1, and IRF7 by multiples of ten magnitudes. The time and degree of this induction mirror the early type I/III IFN response observed in HMPV-infected MDMs. In contrast, RSV provoked a modest increase in ISG15 and IRF7 at 48 h and a negligible induction of OAS1 or MX1. The expression profile of IRF7 was different from the other ISG patterns. Its pronounced upregulation in HMPV-infected macrophages likely reflects autocrine amplification via the early IFN-α/β wave. On the other hand, the delayed increase during RSV infection may be driven by the late IFN-λ1 and IFN-λ2/3 surge shown in Figure 2.

Cytokine secretion frequently diverges from mRNA levels because processes such as alternative splicing, AU-rich element–mediated decay, selective translation, and vesicular export modulate how much protein ultimately reaches the extracellular space [75]. Therefore, we quantified soluble mediators in MDM supernatants to determine how the transcriptional patterns documented in Figure 1 translate into released protein output. HMPV-infected MDMs released substantially more IFN-α, IFN-ω, and IFN-λ than RSV throughout the first 48 h, mirroring the vigorous early burst of type I and III IFN transcripts. The IFN-β protein expression amount was also higher after HMPV infection, yet the difference between both viruses was far smaller than that observed at the mRNA level. That effect could be explained by the potential instability of the mRNA transcripts of IFN-β mediating decay and affecting IFN-β output even when transcripts are abundant [75,76]. Secretion of IFN-γ was not detected in the infected MDMs, suggesting that HMPV has a limited response to induce type II IFNs in MDMs.

However, while HMPV skewed the MDMs response towards an IFN-dominant profile, our data show that RSV triggered a robust proinflammatory profile characterized by increased concentrations of IL-1α, IL-6, TNF-α, and the chemokine CXCL10/IP-10. These IFN-related proteins are classically induced downstream from NF-κB, and their abundance correlated with the stronger NF-κB activation we measured in RSV-infected THP1-Dual macrophages (Figure 6). Because inflammatory mediators act after post-transcriptional checkpoints are cleared [77,78,79], we quantified the secreted protein levels of IL-1α, IL-6, TNF-α, and IP-10. Importantly, each of these mediators is intimately linked to IFN biology: IL-1α can synergize with type I IFNs to enhance ISG transcription [80], IL-6 amplifies STAT1 signaling and participates in macrophages activation [81,82], TNF-α cooperates with IFN-β to drive antiviral gene profiles [83], and CXCL10 is a bona fide IFN-stimulated chemokine that recruits CXCR3^+^ NK and T cells [84,85], thereby extending the reach of macrophage-derived IFN to other effectors of the immune response.

Because RSV-infected MDMs released substantially more IL-1α, IL-6, TNF-α, and CXCL10 than their HMPV-infected counterparts, we next assessed the activity of two key transcription factors using the THP1-Dual™ cells, which report NF-κB activation via secreted alkaline phosphatase and IRF activation via an ISRE-driven luciferase. We used differentiated monocytic THP1-Dual™ cells because they reliably mimic primary monocyte-derived macrophages in morphology, receptor expression, and stimulus-induced cytokine profiles, making them a robust stand-in for mechanistic studies [86,87,88,89,90]. We observed that RSV strongly activates NF-κB in macrophages, while HMPV does not seem to activate it. These findings align with the elevated levels of IL-1α, IL-6, IP-10, and TNF-α detected in the RSV-infected cultures and suggest that NF-κB is a key driver of the RSV-induced inflammatory profile. These data correlate with the observed contribution of alveolar macrophages to the NF-kB response in RSV-infected mice [91]. Likewise, the current observations are congruent with the data reported in MDMs infected with HMPV, where marginal p65 phosphorylation was detected [60,92]. On the other hand, IRF activation was induced by both viruses, which is in line with what has been reported for RSV in a macrophage cell line [93], and in HMPV-infected MDMs [60,92]. We also observed that the IRF activity was unexpectedly higher in RSV-infected cells than in HMPV-infected cells, even though RSV drives a far weaker IFN transcript and protein output. We speculate that the seemingly paradoxical finding that RSV elicits stronger IRF-responsive–luciferase activity in THP1-Dual macrophages while driving only modest IFN gene expression could be explained by the stage and gene-specific actions of the RSV non-structural proteins NS1 and NS2. Early after infection, viral RNA is sensed by RIG-I and MDA5, leading to phosphorylation and the nuclear translocation of IRF3/7; this step is captured efficiently by the ISRE-luciferase reporter, which lacks chromatin restraints and is transcribed as soon as activated IRFs bind its promoter. RSV does not block this initial IRF phosphorylation [64,94], so a high luciferase signal is recorded. Shortly thereafter, however, accumulating NS1/NS2 proteins could inhibit endogenous IFN production that would not affect the plasmid-based reporter. In fact, one of the described mechanisms is that NS1 binds the IRF3 co-activator CBP/p300 without affecting the phosphorylation of IRF3 or translocation, preventing enhanceosome assembly on the IFN-β promoter [64]. However, HMPV, which lacks NS1/NS2, permits the full transcription of IFN genes but actively suppresses NF-κB through its G and SH glycoproteins [94,95]; the resulting cytokine balance is, therefore, IFN-dominant and relatively non-inflammatory, the opposite of the RSV profile. Thus, the differential viral antagonism of NF-κB versus the transcriptional arm of the IRF pathway may explain the distinct cytokine landscapes elicited by these two pneumoviruses.

As we observed, the initial induction of the IFN expression by the viral infections declines over time. That regulation may result from the host cells installing negative feedback loops. For instance, virus-induced SOCS1/3 and the ISG USP18 inhibit the phosphorylation of JAKs and interfere with IFNAR2–STAT2 interactions, respectively, throttling both type I and III IFN signaling cascades [96,97]. Also, high local cytokine concentrations trigger ligand-stimulated endocytosis and the lysosomal degradation of the IFNAR1 subunit (and analogous turnover of IFNLR1), desensitizing cells and accelerating the decay of short-lived IFN mRNAs [98]. Moreover, the viruses could also contribute to that effect, as it has been observed that RSV deploys its non-structural proteins, NS1 and NS2, which both induce SOCS1/3, and NS1 also targets STAT2 for proteasomal degradation, jointly blocking JAK–STAT amplification and shutting down new IFN transcription [99,100,101]. Likewise, HMPV achieves a similar outcome through the M2-2 protein, which binds TRIM25 and prevents RIG-I ubiquitination, thereby aborting MAVS-dependent signaling and curtailing sustained IFN-β/λ gene expression [102].

The observed differences in IFN and inflammatory-cytokine induction between HMPV and RSV in MDMs reflect the innate immune dynamics observed in experimental animal models in vivo [51,55], and in clinical samples from infected infants, in which HMPV induced lower levels of inflammatory cytokines than RSV [103]. Nevertheless, alveolar macrophages are influenced by epithelial crosstalk, local cytokine gradients, and tissue-resident programming. Therefore, to overcome the limitations that a monoculture format entails, such as lacking crosstalk with the airway epithelium and the fact that the MDMs differ in innate-sensor profiles from bona fide alveolar macrophages in the lung, future studies should incorporate airway organoids or macrophage–epithelium co-cultures to capture multicellular interactions.

Clinical HMPV infections are often associated with milder or self-limiting lower-respiratory symptoms compared with those of RSV [104,105,106], suggesting that the stronger type I and III IFN responses induced by HMPV may contribute to more efficient viral clearance and the faster resolution of infection compared to RSV. In contrast, the tendency of RSV to drive stronger inflammatory responses has been linked to enhanced airway pathology, mucus production, and increased hospitalization rates, particularly in infants and the elderly [103,107,108,109,110]. Overall, our findings suggest that the balance between antiviral and inflammatory programs initiated by macrophages may contribute to the differences in disease severity and clinical outcomes between these two pneumoviruses.

## 5. Conclusions

Our work highlights the relevance of macrophages, whose IFN outputs shape the course of pneumovirus infection. HMPV drives an early, robust induction of type I and III IFNs and ISG profiles in infected macrophages while exhibiting a marginal production of proinflammatory cytokines. On the other hand, RSV induces a weak IFN response and skews macrophages toward a proinflammatory cytokine profile. This work demonstrates that macrophages orchestrate virus-specific balances between IFN-driven defense and inflammation during pneumovirus infection, thereby underscoring their central role in respiratory immunity.

## Data Availability

The data supporting the conclusions of this manuscript are all present within the article.
